# Clinical characteristics and a decision tree model to predict death outcome in severe COVID-19 patients

**DOI:** 10.1186/s12879-021-06478-w

**Published:** 2021-08-09

**Authors:** Qiao Yang, Jixi Li, Zhijia Zhang, Xiaocheng Wu, Tongquan Liao, Shiyong Yu, Zaichun You, Xianhua Hou, Jun Ye, Gang Liu, Siyuan Ma, Ganfeng Xie, Yi Zhou, Mengxia Li, Meihui Wu, Yimei Feng, Weili Wang, Lufeng Li, Dongjing Xie, Yunhui Hu, Xi Liu, Bin Wang, Songtao Zhao, Li Li, Chunmei Luo, Tang Tang, Hongmei Wu, Tianyu Hu, Guangrong Yang, Bangyu Luo, Lingchen Li, Xiu Yang, Qi Li, Zhi Xu, Hao Wu, Jianguo Sun

**Affiliations:** 1 Department of Ultrasound, The 941st Hospital of the PLA Joint Logistic Support Force, Xining, People’s Republic of China; 2 Cancer Institute, Xinqiao Hospital, Army Medical University, Chongqing, People’s Republic of China; 3 Department of Clinical Laboratory, Xinqiao Hospital, Army Medical University, Chongqing, People’s Republic of China; 4 Department of Emergency, Xinqiao Hospital, Army Medical University, Chongqing, People’s Republic of China; 5 Xinqiao Hospital, Army Medical University, Chongqing, People’s Republic of China; 6 Department of Cardiology, Xinqiao Hospital, Army Medical University, Chongqing, People’s Republic of China; 7 Department of General Medicine, Xinqiao Hospital, Army Medical University, Chongqing, People’s Republic of China; 8 Department of Neurology, Southwest Hospital, Army Medical University, Chongqing, People’s Republic of China; 9 Department of Gastroenterology, Southwest Hospital, Army Medical University, Chongqing, People’s Republic of China; 10 Pulmonary and Critical Care Medicine Center, Chinese PLA Respiratory Disease Institute, Xinqiao Hospital, Army Medical University, Chongqing, People’s Republic of China; 11 Institute of Burn Research, State Key Laboratory of Trauma, Burns and Combined Injury, Army Medical University, Chongqing, People’s Republic of China; 12 Department of Oncology, Southwest Hospital, Army Medical University, Chongqing, People’s Republic of China; 13 Cancer Center, Army Medical Center, Chongqing, People’s Republic of China; 14 Nursing Department, Army Medical Center, Chongqing, People’s Republic of China; 15 Department of Hematology, Xinqiao Hospital, Army Medical University, Chongqing, People’s Republic of China; 16 Department of Nephrology, The Key Laboratory for the Prevention and Treatment of Chronic Kidney Disease of Chongqing, Kidney Center of PLA, Xinqiao Hospital, Army Medical University, Chongqing, People’s Republic of China; 17 Department of Infectious Diseases, Southwest Hospital, Army Medical University, Chongqing, People’s Republic of China; 18 Department of Neurology, Xinqiao Hospital, Army Medical University, Chongqing, People’s Republic of China; 19 Department of Cardiology, The 958th Hospital, Southwest Hospital, Army Medical University, Chongqing, People’s Republic of China; 20 Department of Gastroenterology, Xinqiao Hospital, Army Medical University, Chongqing, People’s Republic of China; 21 Department of Respiratory Medicine, Army Medical Center, Chongqing, People’s Republic of China; 22 Department of Orthopedics, Xinqiao Hospital, Army Medical University, Chongqing, People’s Republic of China; 23 Department of Obstetrics and Gynecology, Xinqiao Hospital, Army Medical University, Chongqing, People’s Republic of China; 24 Department of Nosocomial Infection Control, Xinqiao Hospital, Army Medical University, Chongqing, People’s Republic of China

**Keywords:** COVID-19, Decision tree, Neutrophil-to-lymphocyte ratio, C-reactive protein, Lactic dehydrogenase

## Abstract

**Background:**

The novel coronavirus disease 2019 (COVID-19) spreads rapidly among people and causes a pandemic. It is of great clinical significance to identify COVID-19 patients with high risk of death.

**Methods:**

A total of 2169 adult COVID-19 patients were enrolled from Wuhan, China, from February 10th to April 15th, 2020. Difference analyses of medical records were performed between severe and non-severe groups, as well as between survivors and non-survivors. In addition, we developed a decision tree model to predict death outcome in severe patients.

**Results:**

Of the 2169 COVID-19 patients, the median age was 61 years and male patients accounted for 48%. A total of 646 patients were diagnosed as severe illness, and 75 patients died. An older median age and a higher proportion of male patients were found in severe group or non-survivors compared to their counterparts. Significant differences in clinical characteristics and laboratory examinations were found between severe and non-severe groups, as well as between survivors and non-survivors. A decision tree, including three biomarkers, neutrophil-to-lymphocyte ratio, C-reactive protein and lactic dehydrogenase, was developed to predict death outcome in severe patients. This model performed well both in training and test datasets. The accuracy of this model were 0.98 in both datasets.

**Conclusion:**

We performed a comprehensive analysis of COVID-19 patients from the outbreak in Wuhan, China, and proposed a simple and clinically operable decision tree to help clinicians rapidly identify COVID-19 patients at high risk of death, to whom priority treatment and intensive care should be given.

**Supplementary Information:**

The online version contains supplementary material available at 10.1186/s12879-021-06478-w.

## Background

The novel coronavirus disease 2019 (COVID-19) has become a pandemic. The most common symptoms of COVID-19 patients were fever, dry cough, fatigue, dyspnea, etc. [1, 2]. A small part of patients had digestive symptoms, such as nausea, vomiting and diarrhea [3, 4]. A study [5] by the Chinese Center for Disease Control and Prevention showed that about 81% COVID-19 patients were considered as mild. The proportion was 14% and 5% respectively, for severe and critical patients, who should be hospitalized or transferred to intensive care unit (ICU) for urgent treatment. The mortality in overall population was 3.2%, but it increased to 49% in critical population. Hence, how to use effective biomarkers to identify patients who are at high risk of poor clinical outcomes have caused extensive concern.

COVID-19 patients with comorbidities were considered to be prone to having poor clinical outcomes. A study revealed that COVID-19 patients with chronic obstructive pulmonary disease, diabetes, hypertension and malignancy had a higher risk of admission to an ICU, invasive ventilation or death [6]. Another study demonstrated that the risk factors included older age, high Sequential Organ Failure Assessment score, and higher D-dimer expression on admission [7].

During the early outbreak of COVID-19 in Wuhan, centre of early stage of the pandemic, medical resources were extremely scarce. It is of great clinical significance to use effective biomarkers to quickly identify patients with high risk of death, to whom should be given priority in accessing medical resources. In this study, we retrospectively enrolled patients from Taikang hospital and other temporary hospitals during the outbreak of COVID-19 in Wuhan, China. We analyzed the differences in clinical characteristics between severe and non-severe patients, as well as survivors and non-survivors. Furthermore, we developed a clinically operable and easy-to-interpret decision tree model to distinguish COVID-19 patients with high risks of death from those without.

## Methods

### Data sources

A total of 2169 adult patients (aged ≥ 18 years) were enrolled from Wuhan, China between February 10th and April 15th, 2020. All patients were confirmed with COVID-19 infection by real-time reverse-transcription polymerase-chain-reaction (RT-PCR) assay. In addition, medical records, including demographics, clinical characteristics and laboratory test results on admission of all patients were also collected. All our data were independent from other hospitals or different in periods from other studies, rather than a repetitive analysis. This study was approved by the Ethics Committee of the Taikang Hospital (TKTJLL-005, TKTJLL-007), and performed in accordance with the Declaration of Helsinki. The Ethics Committee of the Taikang Hospital waived the need for informed consent of each patient. This study was registered in the Clinical Trials Register (NCT04347369, https://clinicaltrials.gov/).

### Study design

First of all, we performed a difference analysis of medical records between severe group and non-severe group. All the patients meeting the severity diagnosis criteria during hospitalization were assigned into the severe group. Disease severity was defined according to the Seventh Revised Trial Version of the COVID-19 Diagnosis and Treatment Guidance (2020) of China [8]. In detail, COVID-19 patients with respiratory rate more than 30 breaths per minute, or oxygen saturation lower than 93% in rest state, or oxygenation index less than 300 mmHg, or rapid progression in lung images within 24–48 h were regarded as severe patients. Next, we performed difference analyses of medical records between survivors and non-survivors. Survivors were defined as patients who were discharged from hospital or transferred to other local hospitals due to advanced age or other basic diseases, instead of COVID-19, at the end of study. Last, we developed a decision tree to predict death outcome.

### Development of a clinically operable decision tree

Many machine learning methods are available to develop a helpful predictive model. However, most of them are difficult to interpret because of their internal model mechanisms of black-box modelling strategies. In this study, we chose the decision tree as the predictive model because it’s visible, clinically operable and easy to interpret due to its recursive tree-based decision system.

Before developing a decision tree, an appropriate data processing is needed. First, laboratory indexes with missing values over 20% were excluded, including interleukin-6 (IL-6), procalcitonin and D-dimer. We also excluded neutrophil count and lymphocyte count but retained neutrophil-to-lymphocyte ratio (NLR) because of a strong correlation. Then all missing values were input with mean value of each remaining laboratory index. Finally, factors including age, sex, smoking status, body temperature, oxygen saturation, heart rate, respiratory rate, number of comorbidities, number of system symptoms, white blood cell (WBC), NLR, monocyte count, eosinophilia count, basophilia count, red blood cell (RBC), hemoglobin, platelet count, lactic dehydrogenase (LDH) and C-reactive protein (CRP) were used in the development of decision tree.

All severe patients were randomly split into training dataset and test dataset with a ratio of 7:3. The training dataset, including 452 severe COVID-19 patients, was used to build the decision tree. And the test dataset, including 194 severe COVID-19 patients, was used to validate the decision tree.

The decision tree is built by a two-stage process and the resulting models can be represented as binary trees. First of all, we explore to find each variable which could best split the data into two groups. The data is separated by related variables recursively until the subgroups either reach a minimum size or until no improvement can be made. The impurity function we used was "Information". In this step, a certain but complex tree model was built. But not all the target variables in the complex model are essential. Hence, secondly, we used cross-validation with the 1-SE rule to trim back the full tree. In the next step, we set the max nodes of split no more than 4 and chose the smallest complexity parameter in order to obtain a simple and meaningful decision tree.

The performance of the model was evaluated by the area under the curve (AUC), accuracy and a confusion matrix which could describe how many results were correctly and incorrectly classified. These indexes were calculated both in the training dataset and the test dataset.

### Statistical analysis

Continuous variables were described as median with interquartile range (IQR), the comparison was analyzed by the Mann–Whitney U test. Categorical variables were represented as frequencies and compared by Pearson’s Χ^2^ test. All statistical analyses were performed and the decision tree model was developed using R software (version 3.5.2). The following R packages were used: CBCgrps, rpart, rpart.plot, MICE and pROC. A two-sided p value < 0.05 was considered statistically significant.

## Results

Of the 2169 COVID-19 patients confirmed by RT-PCR, the median age was 61 years (IQR 50–70; range 18–100 years). Male patients accounted for 48% (1036 cases) and female patients 52% (1133 cases). Approximately 8% of patients (184 cases) had smoking history. On admission, 117 (5%) patients had high body temperature (≥ 37.3 ℃), 270 (12%) had low oxygen saturation (≤ 93%), 359 (17%) had abnormal heart rates and 596 (27%) had faster respiratory rates (> 20 per minute). In total, 1134 (52%) patients had at least one comorbidity, and the common comorbidities were hypertension, diabetes and coronary heart disease. In addition, 728 (34%) patients had one system symptom, 1130 (52%) patients had two system symptoms and 218 (10%) patients had three or more system symptoms. The most common system symptoms were respiratory symptoms, systemic symptoms and digestive symptoms (Table 1).

**Table 1:** Demographics, clinical characteristics and laboratory findings of severe and non-severe COVID-19 patients

Variables	Total (N = 2169)	Severe (N = 646)	Non-severe (N = 1523)	*p* value^a^
Age, years	61 (50, 70)	68 (60, 76)	58 (47, 66)	< 0.001
Sex				< 0.001
Male	1036 (48)	360 (56)	676 (44)	
Female	1133 (52)	286 (44)	847 (56)	
Smoking history				0.582
Never	1985 (92)	587 (91)	1398 (92)	
Former	80 (4)	28 (4)	52 (3)	
Current	104 (4)	31 (5)	73 (5)	
Body temperature				< 0.001
< 37·3 ℃	2052 (95)	589 (91)	1463 (96)	
37.3–38.0 ℃	87 (4)	37 (6)	50 (3)	
> 38.0 ℃	30 (1)	20 (3)	10 (1)	
Oxygen saturation				< 0.001
≤ 93%	270 (12)	270 (42)	0 (0)	
> 93%	1899 (88)	376 (58)	1523 (100)	
Heart rate				0.009
Normal	1810 (83)	518 (80)	1292 (85)	
Abnormal	359 (17)	128 (20)	231 (15)	
Respiratory rate				< 0.001
≤ 20 per minute	1573 (73)	343 (53)	1230 (81)	
21–29 per minute	528 (24)	235 (36)	293 (19)	
≥ 30 per minute	68 (3)	68 (11)	0 (0)	
No. of comorbidities				< 0.001
0	1035 (48)	196 (30)	839 (55)	
1	591 (27)	186 (29)	405 (27)	
≥ 2	543 (25)	264 (41)	279 (18)	
No. of system symptoms				< 0.001
0	93 (4)	12 (2)	81 (5)	
1	728 (34)	164 (25)	564 (37)	
2	1130 (52)	386 (60)	744 (49)	
≥ 3	218 (10)	84 (13)	134 (9)	
White blood cell count, × 10^9^/L	5.8 (4.74, 7.06)	6.23 (4.98, 8)	5.68 (4.69, 6.83)	< 0.001
Neutrophil count, × 10^9^/L	3.48 (2.62, 4.59)	4.02 (3, 5.93)	3.28 (2.52, 4.25)	< 0.001
Lymphocyte count, × 10^9^/L	1.53 (1.13, 1.91)	1.2 (0.8, 1.69)	1.63 (1.27, 1.97)	< 0.001
Neutrophil-to-lymphocyte ratio	2.23 (1.62, 3.31)	3.21 (1.99, 6.62)	2.03 (1.5, 2.77)	< 0.001
Monocyte count, × 10^9^/L	0.47 (0.37, 0.59)	0.48 (0.36, 0.62); n = 646	0.47 (0.38, 0.59); n = 1516	0.906
Eosinophilia count, × 10^9^/L	0.11 (0.06, 0.18)	0.09 (0.03, 0.17); n = 646	0.11 (0.07, 0.18); n = 1516	< 0.001
Basophilia count, × 10^9^/L	0.03 (0.01, 0.04)	0.02 (0.01, 0.03); n = 646	0.03 (0.02, 0.04); n = 1517	< 0.001
Red blood cell count, × 10^9^/L	4.02 (3.63, 4.4)	3.85 (3.44, 4.24); n = 644	4.08 (3.72, 4.46); n = 1513	< 0.001
Hemoglobin, g/L	121 (110, 133)	117 (102, 128); n = 644	123 (113, 134); n = 1514	< 0.001
Platelet count, × 10^9^/L	221.5 (180, 272)	216 (164, 275); n = 645	224 (185, 270); n = 1519	0.008
C-reactive protein, mg/L	1.32 (0.5, 7.35)	6.38 (1, 32.14); n = 638	0.77 (0.5, 3.36); n = 1438	< 0.001
Lactic dehydrogenase, IU/L	176.92 (149.9, 216.95)	212 (172.2, 279.36); n = 625	166.92 (144.97, 197.7); n = 1437	< 0.001
Interleukin-6, pg/mL	2.48 (1.5, 6.36)	6.84 (2.73, 21.23); n = 404	1.77 (1.5, 3.68); n = 961	< 0.001
Procalcitonin, ng/mL	0.05 (0.03, 0.08)	0.07 (0.04, 0.14); n = 514	0.04 (0.03, 0.06); n = 947	< 0.001
D-dimer, μg/mL	0.39 (0.18, 0.8)	0.64 (0.31, 1.49); n = 420	0.3 (0.14, 0.55); n = 701	< 0.001

Data are n (%), n/N (%), or median (IQR), unless specified otherwise. Temperature, oxygen saturation, heart rate and respiratory rate were detected at rest when patients were admitted to hospital

^a^*p* values indicate differences between severe and non-severe patients

A total of 646 (29.8%) patients were diagnosed as severe illness during hospitalization. Compared to non-severe group, severe group had a significantly higher median age (68 vs. 58 years, p < 0.001) and a higher proportion of male patients (56% vs. 44%, p < 0.001). On admission, higher proportions of high body temperature (9%), low oxygen saturation (42%), abnormal heart rate (20%) and faster respiratory rate (47%) were found in severe group. Moreover, patients in severe group had higher proportions of comorbidities (70%) and system symptoms (98%). No difference was found in smoking history (Table 1). When comparing laboratory test results between the two groups, we found that the severe group had significantly higher WBC count, neutrophil count, NLR, CRP, LDH, IL-6, procalcitonin and D-dimer levels, but lower lymphocyte count, eosinophilia count, basophilia count, RBC count, hemoglobin and platelet count. No difference was found in monocyte count (Table 1).

From February 10th to April 15th, 2020, 75 patients died of COVID-19. Differences in demographics and clinical characteristics between survivors and non-survivors were similar to the differences between severe and non-severe groups. For laboratory test comparison, much higher WBC count, neutrophil count, NLR, higher CRP, LDH, IL-6, procalcitonin and D-dimer levels were found in non-survivors (Table 2). RBC count and hemoglobin level showed no difference between the two groups. Other laboratory indexes were lower in non-survivors (Table 2).

**Table 2:** Demographics, clinical characteristics and laboratory findings of survivors and non-survivors

Variables	Non-survivor (N = 75)	Survivor (N = 2094)	*P* value^a^
Age, years	72 (67, 82)	61 (50, 69)	< 0.001
Sex			0.003
Male	49 (65)	987 (47)	
Female	26 (35)	1107 (53)	
Smoking history			0.325
Never	66 (88)	1919 (92)	
Former	3 (4)	77 (4)	
Current	6 (8)	98 (5)	
Body temperature			< 0.001
< 37·3 ℃	54 (72)	1998 (95)	
37.3–38.0 ℃	12 (16)	75 (4)	
> 38.0 ℃	9 (12)	21 (1)	
Oxygen saturation			< 0.001
≤ 93%	59 (79)	211 (10)	
> 93%	16 (21)	1883 (90)	
Heart rate			< 0.001
Normal	51 (68)	1759 (84)	
Abnormal	24 (32)	335 (16)	
Respiratory rate			< 0.001
≤ 20 per minute	26 (35)	1547 (74)	
21–29 per minute	35 (47)	493 (24)	
≥ 30 per minute	14 (19)	54 (3)	
No. of comorbidities			< 0.001
0	11 (15)	1024 (49)	
1	17 (23)	574 (27)	
≥ 2	47 (63)	496 (24)	
No. of system symptoms			0.03
0	3 (4)	90 (4)	
1	14 (19)	714 (34)	
2	49 (65)	1081 (52)	
≥ 3	9 (12)	209 (10)	
White blood cell count, × 10^9^/L	10.2 (7.27, 14.09)	5.75 (4.71, 6.94)	< 0.001
Neutrophil count, × 10^9^/L	9.62 (6.8, 13.53)	3.42 (2.59, 4.46)	< 0.001
Lymphocyte count, × 10^9^/L	0.56 (0.4, 0.8)	1.56 (1.17, 1.92)	< 0.001
Neutrophil-to-lymphocyte ratio	16.98 (11.88, 26.16)	2.18 (1.6, 3.15)	< 0.001
Monocyte count, × 10^9^/L	0.38 (0.22, 0.64); n = 75	0.48 (0.38, 0.59); n = 2087	0.002
Eosinophilia count, × 10^9^/L	0.01 (0, 0.04); n = 75	0.11 (0.06, 0.18); n = 2087	< 0.001
Basophilia count, × 10^9^/L	0.01 (0, 0.02); n = 75	0.03 (0.02, 0.04); n = 2088	< 0.001
Red blood cell count, × 10^9^/L	4.02 (3.31, 4.34); n = 75	4.02 (3.64, 4.4); n = 2083	0.051
Hemoglobin, g/L	118 (101, 134); n = 75	121 (110, 132); n = 2083	0.213
Platelet count, × 10^9^/L	172 (89, 263); n = 75	223 (182, 273); n = 2089	< 0.001
C-reactive protein, mg/L	89.33 (38.26, 135.91); n = 75	1.18 (0.5, 6.09); n = 2001	< 0.001
Lactic dehydrogenase, IU/L	429.14 (366, 541.2); n = 73	175.2 (148.9, 211.2); n = 1989	< 0.001
Interleukin-6, pg/mL	65.28 (20.41, 154.1); n = 27	2.42 (1.5, 5.91); n = 1338	< 0.001
Procalcitonin, ng/mL	0.28 (0.14, 0.66); n = 63	0.04 (0.03, 0.08); n = 1398	< 0.001
D-dimer, μg/mL	2.43 (0.78, 5.61); n = 47	0.37 (0.17, 0.74); n = 1074	< 0.001

Data are n (%), n/N (%), or median (IQR), unless specified otherwise. Temperature, oxygen saturation, heart rate and respiratory rate were detected at rest when patients were admitted to hospital

^a^*p* values indicate differences between survivors and non-survivors

**Fig. 1:**
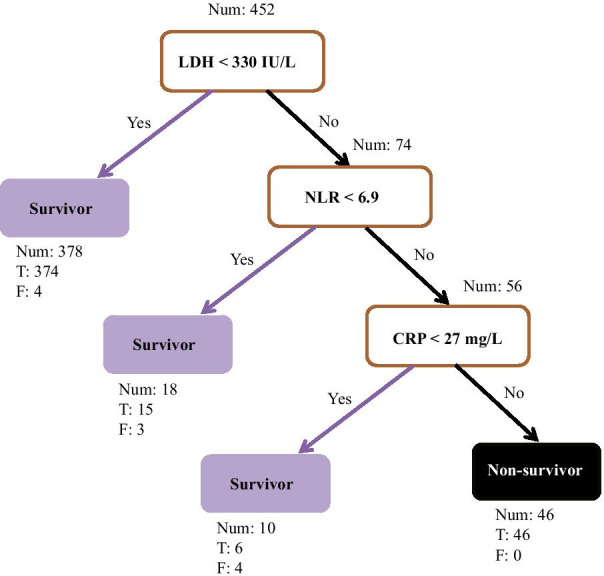
A decision tree model using three biomarkers and their thresholds in absolute value to predict death outcome in severe COVID-19 patients. Num, the number of patients in a class; T, the number of correctly classified patients; F, the number of misclassified patients; NLR, neutrophil-to-lymphocyte ratio; CRP, C-reactive protein; LDH, lactic dehydrogenase; COVID-19, novel coronavirus disease 2019

**Fig. 2:**
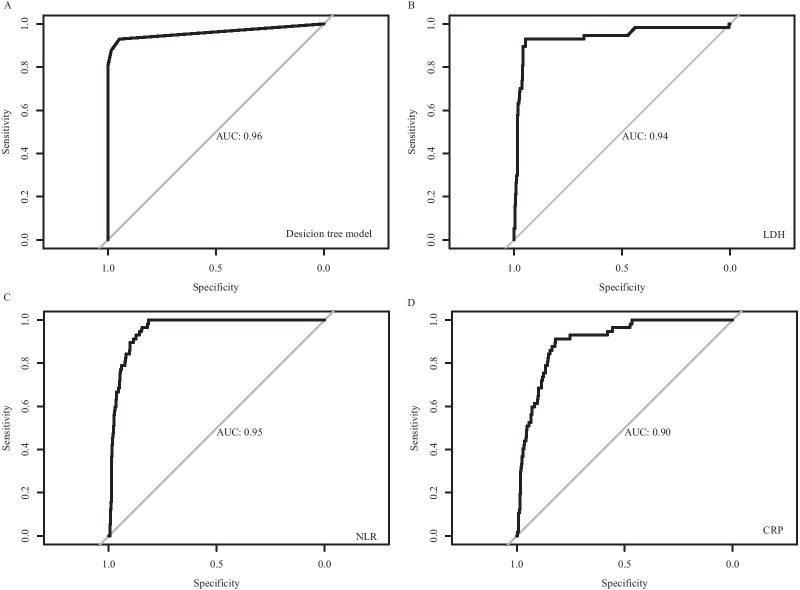
ROC curves for the decision tree model and each biomarker. **A** ROC curve for the decision tree model; **B** ROC curve for LDH; **C** ROC curve for NLR; **D** ROC curve for CRP. ROC, receiver operating characteristic; NLR, neutrophil-to-lymphocyte ratio; CRP, C-reactive protein; LDH, lactic dehydrogenase; AUC, area under the curve of ROC

To explore crucial predictive biomarkers of disease mortality in severe patients, we used a machine learning model, decision tree, to identify related biomarkers. A total of 452 patients were included in the training dataset, including 57 non-survivors. In this step, a decision tree model was developed to differentiate non-survivors from survivors. As shown in Fig. 1, three biomarkers were included in the decision tree model, including LDH, NLR and CRP. The threshold of each biomarker helped to classify each patient into survivor group or non-survivor group. The AUC of the receiver operating characteristic of this model was 0.96, which was higher than each single biomarker (Fig. 2). The associated confusion matrix of training dataset was presented in Additional file 1: Table S1. The accuracy of this model was 0.98. The precision, recall and F1 score for survivor prediction was 0.97, 1.00 and 0.98, respectively. For non-survivors, the precision, recall and F1 score was 1.00, 0.81 and 0.90, respectively (Table 3).

**Table 3:** Performance of the decision tree on the training and test datasets

	Precision	Recall	F1 score	Support
Training dataset				
Survivor	0.97	1.00	0.98	406
Non-survivor	1.00	0.81	0.90	46
Accuracy			0.98	452
Test dataset				
Survivor	0.98	0.99	0.98	178
Non-survivor	0.94	0.83	0.88	16
Accuracy			0.98	194

To validate the performance of the decision tree, we applied it to the test dataset, which included 194 severe patients. The associated confusion matrix of test dataset was presented in Additional file 1: Table S1. The accuracy in test dataset was 0.98. The precision, recall and F1 score for survivor prediction in test dataset was 0.98, 0.99 and 0.98, respectively. For non-survivor prediction in test dataset, the precision, recall and F1 score was 0.94, 0.83 and 0.88, respectively (Table 3).

## Discussion

In this study, we found that COVID-19 patients in severe group or non-survivor group had a higher median age. Also, these patients had higher proportions of comorbidities and symptoms than their counterparts. Zhang et al. [9] reported that the median age in a small cohort of COVID-19 non-survivors was 72.5 years, similar to our findings. In the early outbreak in China, the case fatality ratio (CFR) of COVID-19 was 0.4%, 1.3%, 3.6%, 8% and 14.8% among patients aged 40 s or younger, 50 s, 60 s, 70 s and 80 s or older, respectively [10]. Some studies outside China also showed that the CFR of older patients was much higher than that of younger patients [11–13]. Impairment of immune defense against COVID-19 infection, immunosenescence, and increased risk for immunopathology were thought to be related to higher severity and mortality in older patients [14]. Other proposed hypothesis regarding the vulnerability to COVID-19 among aged patients including age-related chronic inflammation [15] or immunosenescence secondary to cytomegalovirus infection [16, 17]. Fortunately, COVID-19 vaccines might have high efficacy and safety to protect older people from COVID-19 infection [18].

We found that male COVID-19 patients accounted for the majority of severe patients and non-survivors. Previous study also demonstrated that approximately 60% of patients died of COVID-19 were male all over the world [19]. Male had a hazard ratio of 1.59 for COVID-19 related death compared to female [20]. The probable reason might be higher levels of several important proinflammatory innate immune chemokines and cytokines, such as IL-8, IL-18, and CCL5, but weaker T cell response in male patients in comparison with female patients [21]. Besides, behavioral/lifestyle risk factors, prevalence of co-morbidities, aging, and underlying biological sex differences might also contribute to the differences of CFR and severity between male and female patients [22].

Above all, this study proposed a simple and clinically operable decision tree model to quickly quantify the risk of COVID-19 related death based on three biomarkers (LDH, NLR and CRP), which could be easily obtained on admission. Take the training dataset as example (Fig. 1), the first biomarker LDH could divide all 452 patients with severe COVID-19 into two subgroups. Only 4 out of 378 (1.1%) patients with LDH < 330 IU/L died, while 53 out of 74 (71.6%) patients with LDH ≥ 330 IU/L died. Then next biomarker NLR could further stratify the subgroup of LDH ≥ 330 IU/L. Among this subgroup, those with NLR < 6.9 had relatively low risk of death compared to those with NLR ≥ 6.9 (16.7% vs. 89.3%). Moreover, among patients with LDH ≥ 330 IU/L and NLR ≥ 6.9, all those with CRP ≥ 27 mg/L died, 4 out of 10 of those with CRP < 27 mg/L died. In short, we recommend COVID-19 patients with LDH ≥ 330 IU/L and NLR ≥ 6.9 should be closely monitored or transfer to ICU. Those with LDH ≥ 330 IU/L but NLR < 6.9 also need to be carefully observed. This simple decision tree model helps physician quickly identify patients with high risk of death and priority of healthcare should be allocated accordingly, which is especially important in crowed hospital or during COVID-19 outbreak with shortage of medical resources.

Separately, these three biomarkers also have important clinical significance. The increase of LDH is a marker of tissue/cell damage. In patients with idiopathic pulmonary fibrosis, the LDH level could reflect the extent of lung injury [23]. For patients with severe COVID-19, the rise in LDH might indicate the activity of lung injury. Evidence proved that LDH was a biomarker of severe illness and poor prognosis in COVID-19 patients [24]. Zeng et al. found that LDH decreased within 10 days after admission in non-critical COVID-19 patients, but did not decrease obviously in critical patients or non-survivors [25]. NLR is one of the research hotspots of inflammatory biomarkers in infectious diseases. It can comprehensively reflect the inflammatory response and immune status in patients with infectious diseases [26–28]. In COVID-19 patients, elevated NLR on admission was reported to be significantly associated with disease severity [29, 30]. Liu and colleagues proposed a simple model based on NLR and age to stratify COVID-19 patients into four groups [31]. COVID-19 patients with age < 50 years and NLR < 3.13 or NLR ≥ 3.13 had no risk of severity, and these patients should be treated in a community hospital, home isolation or general isolation ward. While COVID-19 patients with age ≥ 50 and NLR < 3.13 or NLR ≥ 3.13 had a higher risk of severity, and these patients should be admitted to isolation ward or ICU with active treatment and care. In addition, Yang and coworkers found that approximately 46.1% of the mild COVID-19 patients could become severely ill in patients with age ≥ 49.5 and NLR ≥ 3.3 [30]. The dynamic change of NLR could also be used to distinguish severe patients from mild/moderate patients. A study demonstrated that NLR in severe group always kept a higher level on day 1, 4 and 14 compared with mild/moderate group [32]. CRP reflects a persistent inflammatory activity state, and helps in assessing the severity of infectious patients [33]. A few studies have demonstrated that a higher CRP expression on admission was observed in severe COVID-19 patients compared with non-severe COVID-19 patients [33, 34].

Some certain limitations should be acknowledged in this study. First, because of the limited data source, an external validation needs to be performed in further studies. Second, the dynamic changes of some important biomarkers should be followed up to better and timely identify patients at higher risks of death. Third, because some markers, such as IL-6, procalcitonin, D-dimer, etc. were not enough in the study, further study should consider more markers in the development of decision tree.

## Conclusion

In summary, this study found that male COVID-19 patients were more prone to experience severe illness and death. Clinical characteristics and laboratory examinations were significantly different between severe and non-severe groups, as well as between survivors and non-survivors. Most importantly, we proposed a simple, clinically operable and easy-to-interpret decision tree based on three biomarkers (LDH, NLR and CRP) on admission which could easily be obtained in clinical, to help clinicians rapidly identify COVID-19 patients at high risks of death, to whom priority treatment and intensive care should be given.

## Supplementary Information

**Additional file 1: Table S1.** Confusion matrixes of train and test datasets in the decision tree model

## Data Availability

The datasets used and/or analysed during the current study are available from the corresponding author on reasonable request.

## Abbreviations

COVID-19: Novel coronavirus disease 2019
ICU: Intensive Care Unit
RT-PCR: Real-time reverse-transcription polymerase-chain-reaction
IQR: Interquartile range
WBC: White blood cell
NLR: Neutrophil-to-lymphocyte ratio
CRP: C-reactive protein;
LDH: Lactic dehydrogenase
IL-6: Interleukin-6
RBC: Red blood cell
AUC: Area under the curve
