# Supplementary material for: Clinical characteristics and a decision tree model to predict death outcome in severe COVID-19 patients
Source: BMC Infect Dis. 2021 Aug 9;21:783. doi: 10.1186/s12879-021-06478-w (PMC8351764; doi:10.1186/s12879-021-06478-w)
Supplement: Supplementary file 1 — Additional file 1: Table S1. Confusion matrixes of train and test datasets in the decision tree model [file 12879_2021_6478_MOESM1_ESM.docx]

**Supplementary Table 1: Confusion matrixes of train and test datasets in the decision tree model**

| **Train dataset** | Predicted Positive | Predicted Negative |
| --- | --- | --- |
| Real Positive | 46 | 11 |
| Real Negative | 0 | 395 |
| **Test dataset** | Predicted Positive | Predicted Negative |
| Real Positive | 15 | 3 |
| Real Negative | 1 | 175 |
